# Partially covered metal stents have longer patency than uncovered and fully covered metal stents in the management of distal malignant biliary obstruction: a retrospective study

**DOI:** 10.1186/s12876-017-0662-1

**Published:** 2017-10-11

**Authors:** Yudai Yokota, Mitsuharu Fukasawa, Shinichi Takano, Makoto Kadokura, Hiroko Shindo, Ei Takahashi, Sumio Hirose, Satoshi Kawakami, Yoshimitsu Fukasawa, Tadashi Sato, Nobuyuki Enomoto

**Affiliations:** 1 First Department of Internal Medicine, Faculty of Medicine, University of Yamanashi, 1110, Shimokato, Chuo, Yamanashi, 409-3898 Japan; 2 Department of Gastroenterology, Kofu Municipal Hospital, 366, Masutsubo, Kofu, Yamanashi, 400-0832 Japan

**Keywords:** Covered biliary metal stent, Malignant distal biliary obstruction, Stent migration

## Abstract

**Background:**

Self-expandable metal stents (SEMSs) are widely used for malignant biliary obstructions. Nitinol-covered SEMSs have been developed to improve stent patency. Currently, SEMSs may be uncovered, partially covered, or fully covered; however, there is no consensus on the best stent type for the management of malignant distal biliary obstruction (MDBO).

**Methods:**

Patients with unresectable MDBO receiving SEMS (Wallflex™) were retrospectively analyzed. Time to recurrent biliary obstruction (TRBO) and survival time were compared among the three types of SEMSs. Univariate and multivariate analyses were performed to identify risk factors for stent dysfunction.

**Results:**

In total, 101 patients received SEMSs for unresectable MDBO (44 uncovered, 28 partially covered, and 29 fully covered SEMSs). Median survival time was 200, 168, and 276 days in the uncovered, partially covered, and fully covered SEMSs groups, respectively. There were no differences in survival among the three groups. Median TRBO was 199, 444, and 194 days in the uncovered, partially covered, and fully covered SEMSs groups, respectively. Partially covered SEMSs had longer TRBO than uncovered (*p* = 0.013) and fully covered (*p* = 0.010) SEMSs. Tumor ingrowth occurred only with uncovered SEMSs and stent migration occurred only with fully covered SEMSs. Multivariate analyses confirmed that partially covered SEMSs have lower risk of dysfunction.

**Conclusions:**

Partially covered SEMSs with a proximal uncovered flared end have longer patency than uncovered and fully covered SEMSs by preventing tumor ingrowth and stent migration.

## Background

Patients with cancer of the pancreatic head or bile duct are often diagnosed with advanced disease, which is usually unresectable. These patients require adequate palliative treatment for malignant biliary obstructions. Endoscopic stent insertion has become an established procedure for the management of obstructive jaundice of these patients [1].

Self-expandable metal stents (SEMSs) were introduced at the end of the 1980s to improve biliary endoprosthesis patency. SEMSs expand to a larger diameter than plastic stents after placement, and many studies have demonstrated the superiority of stent patency in SEMSs compared with plastic stents [2–7]. However, SEMSs were more prone to occlusion than plastic stents, mainly by tumor ingrowth through the mesh. To overcome this issue, covered SEMSs, in which the stent mesh was covered by a thin membrane, were developed in the 1990s. Several randomized studies have compared the patency of covered SEMSs with that of uncovered SEMSs [8–16]. Some of these studies demonstrated the superiority of covered SEMSs to uncovered SEMSs, whereas others did not. Similarly, two meta-analyses of these studies and retrospective cohort studies have different conclusions [17–23].

The Wallflex™ Biliary RX stent (Boston Scientific Corp, Natick, Mass, USA) was introduced recently in clinical practice [24–26]. This SEMS is different from the previous Wallstent™ model in some areas. Although both stents have a braided structure, Wallflex™ is constructed with nitinol wire, which gives the stent lower axial force, whereas the Wallstent™ is made of stainless wire and has looped and flared ends designed to decrease the risk of tissue trauma and stent migration. There are three types of Wallflex™ stents: uncovered, partially covered, and fully covered, all with the same flared ends. The partially covered stent is covered with a silicone membrane except for 5-mm sections on either end, and the fully covered is almost completely covered, except for a 2-mm segment at the distal end. To date, no study has compared these two SEMSs.

The aim of our study was to compare the clinical outcome of uncovered, partially covered, and fully covered SEMSs for palliation of patients with malignant distal biliary obstruction (MDBO). Factors associated with recurrent biliary obstruction were also evaluated.

## Methods

### Patients

This retrospective analysis included consecutive patients with unresectable MDBO who underwent SEMS placement between May 2009 and July 2014 at Yamanashi University Hospital and Kofu Municipal Hospital. All patients included in the present study had undergone transpapillary insertion of Wallflex™ uncovered, partially covered, or fully covered biliary stents. Patients whose SEMS distal end was placed in the common bile duct were excluded. The diagnosis of malignancy was based on pathological and/or typical radiological findings. This retrospective study was approved by the Human Ethics Review Committee of Yamanashi University Hospital. Informed consent about study participation was officially announced on a web page.

### Procedures

When patients presented with obstructive jaundice, endoscopic retrograde cholangiopancreatography (ERCP) was carried out for biliary drainage and/or cytological diagnosis. After confirmation of unresectable MDBO, an uncovered, partially covered, or fully covered SEMS (Wallflex™ Biliary RX stent; Boston Scientific Corporation) was deployed at the biliary stricture. All SEMSs were 10-mm in diameter. With cases of obvious unresectability and malignancy of the disease, endoscopic SEMS placement was sometimes undertaken without prior biliary drainage on the basis of clinical history or radiological findings. We routinely perform sphincterotomy before SEMS insertion except when the tumor has invaded the papilla and/or the patient has hemorrhagic diathesis. SEMS length was based on the anatomic circumstances and stricture length. The distal end of the SEMS was placed in the duodenal lumen to protrude from duodenal wall for approximately 1 cm. Prophylactic antibiotics and protease inhibitors were routinely given prior to ERCP.

### Definitions

All the terms used in this study follow TOKYO criteria 2014 for transpapillary biliary stenting [27]. Stent occlusion was defined as the presence of clinical features suggestive of obstructive jaundice or cholangitis, or when imaging studies showed insufficient biliary dilation. If ERCP was carried out, stent occlusion was confirmed by cholangiography and the cause of stent obstruction was identified; otherwise, the cause of stent occlusion was considered unknown. Stent migration was defined as dislocation of the stent on radiological or endoscopic examinations. Both stent occlusion and stent migration that required re-intervention were considered as recurrent biliary obstruction. Time to recurrent biliary obstruction (TRBO) was also defined according to TOKYO criteria 2014 as the time interval between initial placement and recurrent biliary obstruction. Survival time was defined from the time of stent insertion to death or last follow-up. Complications other than recurrent biliary obstruction were also reported according to TOKYO criteria 2014.

### Statistical analysis

Patient characteristics and stent adverse events were reported using median and range for continuous variables and counts with proportions for categorical variables. Continuous variables were compared across the three groups using the Kruskal–Wallis test and categorical variables using χ^2^ or Fisher’s exact test, as appropriate.

The main outcome under investigation was TRBO. Patients not experiencing recurrent biliary obstruction were censored at the time of last follow-up or the time of death. SEMS removed because of other stent-related complications were also censored at the time of SEMS replacement.

TRBO and survival time were estimated using the Kaplan–Meier technique and supplemented by the log-rank test for comparisons among the groups. TRBO and survival time were reported as 50% patent periods and 50% survival periods, respectively. In addition, non-obstruction rates at 3, 6, and 12 months estimated using Kaplan–Meier technique were reported.

Univariate analysis was used to assess the prognostic value of related clinical variables, such as type of stent, patient age, sex, type of primary malignancy, location of stricture, length of stricture, length of the portion of the stent over the stricture, prior transpapillary drainage, history of cholecystectomy, endoscopic sphincterotomy prior to stent placement, presence of duodenal invasion, presence of duodenal stent, and antitumor treatment. Variables with a *P* value of <0.20 were included into a multivariate Cox regression analysis to estimate an adjusted hazard ratio (HR) with 95% confidence intervals (CIs).

A *P* value of <0.05 was considered to be statistically significant. All statistical analyses were performed using PASW Statistics 18 (formerly SPSS statistical software) (Tokyo, Japan).

## Results

### Patient characteristics

A total of 101 patients were included the present study. Of these, 44 received uncovered SEMSs, 28 received partially covered SEMSs, and 29 received fully covered SEMSs. Detailed patient characteristics are shown in Table 1. There was no difference among the three groups in terms of age, performance status, cause of stricture, length of stricture, length of the portion of the stent over the stricture, prior transpapillary drainage, sphincterotomy prior to stent placement, history of cholecystectomy, ascites, chemotherapy administration, presence of duodenal invasion, duodenal stent, follow-up period, and patient outcome. However, there were significantly more women in the uncovered SEMSs group than in the other two groups (*p* = 0.003). In addition, 8-cm stents were used significantly more often in the uncovered SEMSs group than in the other two groups (*p* = 0.011). There were significantly fewer patients whose stricture was at the middle of the bile duct in the fully covered SEMSs group (*p* = 0.023). When only patients with pancreatic cancer were analyzed (*n* = 80), there were also significant differences in sex and stent length among the three groups, but there was no significant difference among patients with stricture at the middle of bile duct (*p* = 0.098).

**Table 1:** Patient characteristics

	Uncovered		Partially covered		Fully covered		
	(*n* = 44)		(*n* = 28)		(*n* = 29)		*P* value
Age	75	(54–92)	76	(48–91)	72	(47–87)	0.304
Sex (Male/Female)	18/26		19/9		23/6		0.003*
Performance Status (≤2/3≤)	40/4		24/4		29/0		0.103
Diagnosis							
Pancreas cancer	34	(77.3)	21	(75.0)	25	(86.2)	
Bile duct cancer	6	(13.6)	4	(14.3)	2	(6.9)	>0.5
Other	4	(9.1)	3	(10.7)	2	(6.9)	
Stent length							
4 mm	1	(2.3)	0		1	(3.5)	
6 mm	32	(72.7)	27	(96.4)	27	(93.0)	0.011*
8 mm	11	(25.0)	1	(3.6)	1	(3.5)	
Stricture location							
Middle	14	(31.8)	9	(32.1)	2	(7.0)	0.023*
Lower	30	(68.2)	19	(67.9)	27	(93.0)	
Length of stricture (mm)	22	(8–100)	20	(7–55)	25	(10–52)	0.218
Length of a portion of the stent over the stricture (mm)	30	(5–70)	27.5	(7–55)	25	(0–60)	0.159
Prior transpapillary drainage	28	(63.6)	13	(46.4)	19	(65.5)	0.255
Sphincterotomy	35	(79.5)	26	(92.9)	25	(86.2)	0.355
History of cholecystectomy	3	(6.8)	2	(7.1)	2	(6.9)	>0.5
Ascites	12	(27.3)	6	(21.4)	6	(20.7)	>0.5
Chemotherapy	29	(65.9)	18	(64.3)	24	(82.8)	0.218
Duodenal invasion	13	(29.5)	7	(25.0)	14	(48.3)	0.132
Duodenal stent	6	(13.6)	4	(14.3)	6	(20.7)	>0.5
Follow-up (days)	172	(30–504)	161	(42–1401)	238	(32–554)	0.243
Outcome (dead/alive)	33/11		25/3		23/6		0.354

*Statistically significant at *p* < 0.05

Pancreatic cancer was the most common primary malignancy, accounting for 79.2% (n = 80) of the study population. Bile duct cancer, including intrahepatic and extrahepatic cholangiocarcinoma, gall bladder cancer, and ampullary cancers, were present in 11.9% (*n* = 12) of all patients. Other primary malignancy included metastatic lymph node (5.0%, *n* = 5), metastatic pancreatic tumor (3.0%, *n* = 3), and pancreatic neuroendocrine carcinoma (1.0%, *n* = 1).

### Technical and functional success

All patients had successful deployment of SEMSs. In addition, functional success was achieved in all patients.

### Patient survival

**Fig. 1:**
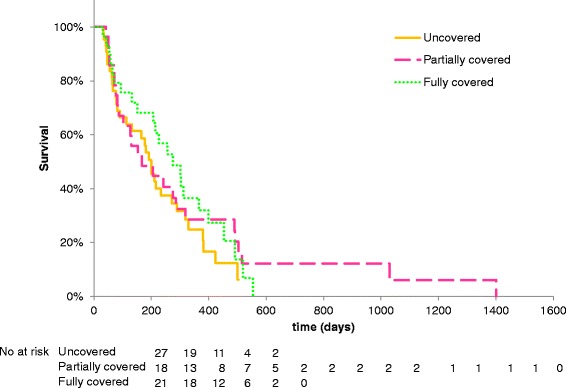
Kaplan–Meier graph showing survival among the three groups. There were no significant differences between uncovered vs partially covered (*p* = 0.482); partially covered vs fully covered (*p* = 0.928); and fully covered vs uncovered (*p* = 0.203) groups

Median follow-up after stent placement was 172, 161, and 238 days for the uncovered, partially covered, and fully covered groups, respectively. There was no significant difference in follow-up periods among the three groups (Table 1). Kaplan–Meier survival analysis showed no significant difference in survival time among the three groups (Fig. 1). Median survival time was 200, 168, and 276 days in the uncovered, partially covered, and fully covered groups, respectively.

### Stent patency analysis

**Fig. 2:**
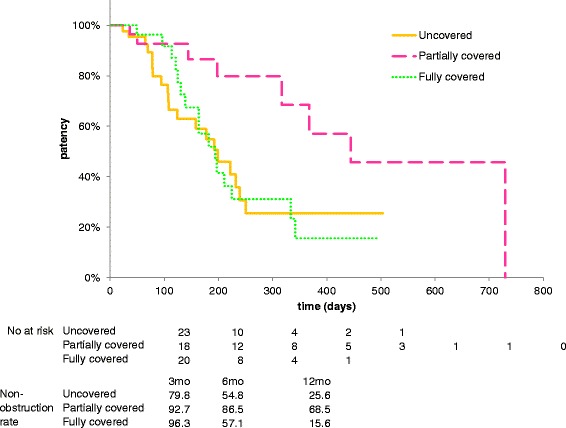
Kaplan–Meier graph showing stent patency among the three groups. The cumulative time to stent dysfunction or patient death was significantly longer in the partially covered group then the other two groups (vs uncovered, *p* = 0.013; vs fully covered, *p* = 0.010). There was no significant difference between the uncovered group and the fully covered group (*p* = 0.830)

Median TRBO was 199, 444, and 194 days for uncovered, partially covered, and fully covered SEMS groups, respectively (Fig. 2). Non-obstruction rates at 3, 6, and 12 months were 79.8%, 54.8% and 25.6% in the uncovered group, 92.7%, 86.5%, and 68.5% in the partially covered group, and 96.3%, 57.1%, and 15.6% in the fully covered group, respectively. TRBO of partially covered SEMSs was significantly longer than that of the other two groups (versus uncovered, *p* = 0.013; versus fully covered, *p* = 0.010). When only patients with pancreatic cancer (*n* = 80; 34 in the uncovered, 21 in the partially covered, and 25 in the fully covered group) was analyzed, partially covered SEMSs also had longer TRBO than the other two stents (versus uncovered, *p* = 0.006; versus fully covered, *p* = 0.008).

The causes of recurrent biliary obstruction are shown in Table 2. Recurrent biliary obstruction occurred in 20 patients (45.4%) in the uncovered group, eight (28.6%) in the partially covered group, and 16 (55.2%) in the fully covered group. Crushed stents or inadequate stent expansion was not observed in this study. No tumor ingrowth occurred in the partially and fully covered groups, although it was observed in 11 (25.0%) of the uncovered group (*p* < 0.001). When the SEMS was occluded by tumor ingrowth or overgrowth, a new SEMS was placed in the occluded stent. When the SEMS occluded by food impaction or sludge, stent cleaning was performed.

**Table 2:** Causes of recurrent biliary obstruction

	Uncovered		Partially covered		Fully covered		
	(*n* = 44)		(*n* = 28)		(*n* = 29)		*P* value
Recurrent biliary obstruction	20	(45.4%)	8	(28.6%)	16	(55.2%)	0.122
Tumor ingrowth	11	(25.0%)	0		0		<0.001*
Tumor overgrowth	1	(2.3%)	1	(3.6%)	2	(6.9%)	0.61
Sludge	2	(4.5%)	3	(10.7%)	1	(3.4%)	0.489
Stent migration	0		0		9	(31.0%)	<0.001*
Unknown	6	(13.6%)	4	(14.3%)	4	(13.8%)	1.00

*Statistically significant at *p* < 0.05

Stent migration was observed in 9 patients, all in the fully covered group (31.0%). No stent migration occurred in the uncovered and partially covered groups. All of these stents migrated distally. Of these stents, seven dropped into the digestive tract and two stents migrated partially with the proximal part of the stents retained in the bile duct. Five patients whose stents dropped out presented with obstructive jaundice because of stent migration and a new metallic stent was placed. In one patient, the migrated stent abutted the opposite wall of duodenum and led to traumatic duodenal perforation. One patient demonstrated the absence of the SEMS on abdominal computed tomography and was diagnosed with stent migration.

The two partially migrated stents were removed endoscopically and replaced with a new metallic stent.

### Risk factors for recurrent biliary obstruction

The univariate and multivariate analyses of the risk factors for recurrent biliary obstruction are shown in Table 3. Univariate analysis revealed that only partially covered SEMSs were significantly at less risk of stent dysfunction (*p* = 0.012). Type of SEMSs, sex, performance status, primary malignancy, length of the stricture, stent length, duodenal invasion, and chemotherapy administration were included in the multivariate analysis because their associated *P* values were <0.20 in univariate analysis. There were three independent factors for recurrent biliary obstruction: partially covered SEMSs (HR = 0.296, 95% CI 0.111–0.774, *p* = 0.013), pancreatic cancer (HR 3.759, 95% CI 1.098–12.866, *p* = 0.035), and performance status (HR = 4.907, 95% CI 1.125–21.398, *p* = 0.034). When the same analysis was performed in patients with pancreatic cancer (Table 4), performance status was no longer a significant factor whereas the partially covered SEMS was the only independent good predictor for stent patency (HR = 0.325, 95% CI 0.123–0.858, *p* = 0.023).

**Table 3:** Univariate and multivariate analyses of risk factors for recurrent biliary obstruction

			Stent dysfunction (*n* = 44)	univariate			multivariate		
		*n*		HR	95% CI	*P* value	HR	95% CI	*P* value
Type of stent	Uncovered	44	20			1			
	Partially	28	8	0.33	0.14–0.79	0.012*	0.30	0.11–0.77	0.013*
	Fully	29	16	0.96	0.49–1.85	0.892	0.92	0.43–1.95	0.822
Age	<75	48	20			1			
	≥75	53	24	1.25	0.68–2.30	0.464			
Sex	male	41	23			1			
	female	60	21	1.54	0.84–2.80	0.162	1.50	0.75–3.03	0.256
Performance status	0–2	93	41			1			
	3,4	8	3	3.14	0.93–10.60	0.066	4.91	1.13–21.40	0.034*
Primary malignancy	others	21	3			1			
	pancreatic	80	41	2.89	0.89–9.35	0.077	3.76	1.10–12.87	0.035*
location of stricture	lower	76	34			1			
	middle	25	10	0.64	0.31–1.35	0.241			
Length of stricture	≤20 mm	53	20			1			
	>20 mm	48	24	1.61	0.88–2.95	0.120	1.33	0.69–2.54	0.395
Stent length	≤6 cm	88	39			1			
	8 cm	13	5	2.24	0.87–5.77	0.095	2.16	0.73–6.38	0.165
Length of a portion of the stent over the stricture	<30 mm	49	23			1			
	≥30 mm	52	21	1.09	0.59–1.99	0.787			
Prior transpapillary Drainage	No	41	15			1			
	Yes	60	29	0.72	0.38–1.36	0.310			
History of cholecystectomy	No	94	43			1			
	Yes	7	1	0.62	0.08–4.53	0.638			
EST	No	14	7			1			
	Yes	87	37	0.72	0.30–1.72	0.464			
duodenal invasion	absent	67	22			1			
	present	34	22	1.68	0.92–3.06	0.091	1.62	0.81–3.25	0.172
Duodenal stent	absent	85	35			1			
	Present	16	9	1.29	0.60–2.79	0.518			
Chemotherapy	No	30	9			1			
	Yes	71	35	0.55	0.26–1.17	0.120	0.47	0.19–1.15	0.098

*HR* Hazard Ratio, *CI* Confidence Interval, *EST* endoscopic sphincterotomy

*Statistically significant at *p* < 0.05

**Table 4:** Univariate and multivariate analyses of risk factors for recurrent biliary obstruction in pancreatic cancer

			Stent dysfunction (*n* = 41)	Univariate			Multivariate		
		*n*		HR	95% CI	*P* value	HR	95% CI	*P* value
Type of stent	Uncovered	34	18	1					
	Partially	21	7	0.27	0.11–0.69	0.006*	0.33	0.12–0.86	0.023*
	Fully	25	16	0.87	0.44–1.71	0.687	0.88	0.43–1.78	0.719
Age	<75	40	20	1					
	≥75	40	21	1.05	0.56–1.97	0.874			
Sex	Male	48	22	1					
	Female	32	19	1.67	0.89–3.12	0.111	1.62	0.82–3.19	0.163
Performance status	0–2	75	39	1					
	3,4	5	2	2.22	0.52–9.49	0.283			
Location of stricture	Lower	62	31	1					
	Middle	18	10	0.69	0.33–1.45	0.327			
Length of stricture	≤20 mm	42	18	1					
	>20 mm	38	23	1.71	0.92–3.20	0.092	1.33	0.67–2.64	0.418
Stent length	≤6 cm	72	37	1					
	8 cm	8	4	2.33	0.81–6.69	0.117	2.26	0.70–7.31	0.172
Length of a portion of the stent over the stricture	<30 mm	36	22	1					
	≥30 mm	44	19	0.92	0.49–1.72	0.787			
Prior transpapillary drainage	No	33	13	1					
	Yes	47	28	0.96	0.49–1.88	0.906			
History of cholecystectomy	No	75	40	1					
	Yes	5	1	0.60	0.08–4.36	0.610			
EST	No	8	5	1					
	Yes	72	36	0.95	0.34–2.68	0.921			
Duodenal invasion	absent	49	21	1					
	present	31	20	1.27	0.68–2.36	0.460			
Duodenal stent	absent	65	32	1					
	Present	15	9	1.12	0.52–2.44	0.770			
Chemotherapy	No	19	7	1					
	Yes	61	34	0.59	0.26–1.35	0.211			

*HR* Hazard Ratio, *CI* Confidence Interval, *EST* Endoscopic Sphincterotomy

*Statistically significant at *p* < 0.05

### Complications other than recurrent biliary obstruction

Complications other than recurrent biliary obstruction are listed on Table 5. Overall complication rates were not significantly different among the three groups. Acute pancreatitis occurred in 10 patients (nine had mild pancreatitis and one had moderate pancreatitis) the day after stent placement across the three groups. Of these, all patients with the exception of one in the uncovered group were treated conservatively. One patient with uncovered SEMS showed a dilated pancreatic duct, suggesting obstructive pancreatitis. This uncovered SEMS was removed and replaced with a new uncovered SEMS 2 days after the first SEMS placement, of which the distal end was placed in the common bile duct so as not to compress the orifice of pancreatic duct. Moderate cholecystitis occurred in one of the partially covered and two of the fully covered group. The patient with the partially covered SEMS developed cholecystitis 1 day after SEMS placement, and the two patients with the fully covered SEMS developed cholecystitis 10 days and 37 days, respectively, after SEMS placement. They underwent percutaneous gall bladder drainage and improved rapidly, and the covered stents were not removed. Mild cholangitis occurred in two patients in each of the groups. They received oral and/or intravenous antibiotics and recovered without further intervention. One patient in the fully covered group developed anemia and presented with melena 4 days after stent placement. He was on anticoagulation therapy for his pulmonary embolism. Endoscopic examination revealed postsphincterotomy bleeding and he was successfully treated endoscopically. He did not require a transfusion. There were no procedure-related severe adverse events or mortality.

**Table 5:** Complications other than recurrent biliary obstruction

	Uncovered		Partially covered		Fully covered		
	(*n* = 44)		(*n* = 28)		(*n* = 29)		*P* value
Pancreatitis	5	(11.3%)	3	(10.7%)	2	(6.9%)	0.81
Cholecystitis	0		1	(3.6%)	2	(6.9%)	0.176
Cholangitis without stent occlusion	2	(4.5%)	2	(7.1%)	2	(6.9%)	0.87
Hemorrhage	0		0		1	(3.4%)	0.29
Total	7	(15.9%)	6	(21.4%)	7	(24.1%)	0.67

## Discussion

This is the first study comparing TRBO and complications among the three types of SEMSs (uncovered, partially covered, and fully covered). All SEMS were constructed of same material and configuration (Wallflex™); the stent covering was the only difference among the three types. Although it did not differ significantly, the recurrent biliary obstruction rate of partially covered SEMS (28.6%) was lower than that of uncovered (45.4%, *p* = 0.215) and fully covered (55.2%, *p* = 0.106) stents. Tumor ingrowth was observed significantly more frequently in the uncovered group (25.0%), and stent migration was observed significantly more frequently in the fully covered group (31.0%). These complications were not observed in the partially covered group. Accordingly, the partially covered group had longer TRBO than the other two groups and multivariate analysis demonstrated that the use of partially covered stents was an independent factor to decrease the risk of recurrent biliary obstruction.

SEMS are shown to have longer stent patency than plastic stents in malignant biliary obstruction; however, tumor ingrowth through the mesh is a problem with uncovered SEMSs. To overcome this problem, covered SEMSs were developed to prevent tumor ingrowth and, in the majority of previous studies, occurrence of tumor ingrowth in covered SEMSs was significantly lower than that of uncovered SEMSs [8, 10–13, 16, 19, 21]. On the other hand, stent migration was more frequently observed in covered SEMSs in some previous studies and was regarded as a major cause of recurrent biliary obstruction [9, 10, 17–19, 22, 28]. Six previous studies, which failed to prove the superiority of covered versus uncovered SEMSs in TRBO, showed higher stent migration rate in the covered group than in the uncovered group [9, 10, 18–20, 22]. In contrast, in the other studies that demonstrated longer TRBO using covered SEMSs, stent migration of covered SEMSs was rarely observed [8, 11–13, 21]. These results suggest stent migration mostly affects covered SEMSs.

The risk of stent migration is related to the conformability of the stent in the bile duct, which is influenced by the axial force, the recovery force that leads to an SEMS straightening after being bent. SEMS with high axial force and low flexibility are known to increase the risk of stent-related complications, including stent migration and bile duct kinking [29–34]. Isayama et al. [32] and Nakai et al. [34] measured the axial force of a variety of commercially available biliary SEMSs, including the SEMSs in some of the aforementioned studies. SEMSs with high axial force had a higher rate of migration, ranging from 5.6% to 8.8% [9, 19, 20], whereas SEMSs with low axial force had a lower migration rate (1.8% [8] and 0% [13]). Covered SEMSs with low axial force decrease the risk of stent migration, leading to a longer TRBO.

The Wallflex™ biliary stent has a relatively low axial force and is designed with both ends flared to decrease the risk of stent migration. There are three types of Wallflex™ stent with respect to covering: an uncovered stent, a partially covered stent (5-mm uncovered flared portions at either end), and the fully covered stent, which is totally covered except for a 2-mm uncovered flared portion at the distal end. A recent randomized controlled trial using Wallflex™ biliary stent reported that partially covered SEMSs had a longer duration of stent patency than uncovered SEMSs, and no stent migration. To our knowledge, there is no study that compares partially covered and fully covered SEMSs. In the present study, consistent with recent reports, there was no stent migration in the uncovered and partially covered SEMS groups; whereas nine patients (31.0%) in the fully covered SEMS group had stent migration. Although fully covered Wallflex™ stents has the same flared end structures as uncovered and partially covered SEMSs, the proximal flared end is completely covered with silicone membrane. Therefore, the covered flared end could not be embedded in bile duct and failed to work as an anti-migration system.

Krokidis et al. [11, 12] compared Viabil covered biliary SEMSs with uncovered SEMSs. This fully covered SEMS is unique in that multiple sections of the wires near each end of the nitinol stent project outward from the external surface of the tubular lining and act as anchoring fins. Although these SEMSs were not inserted through an endoscopic transpapillary approach, the authors demonstrated the superiority of covered SEMSs in terms of stent patency and no stent migration in covered SEMSs in their two randomized controlled studies, even though the stents were fully covered. Anti-migration systems, such as uncovered flared ends or anchoring fins, can prolong patency of covered SEMSs.

Multivariate analysis revealed that pancreatic cancer was an independent factor for recurrent biliary obstruction. Pancreatic cancer has a more aggressive behavior and poorer prognosis than the other causative tumors. It tends to infiltrate from outside of the bile ducts, whereas bile duct cancer tends to spread linearly along the ducts. The biological nature of pancreatic cancer may explain this result. Because risk of recurrent biliary obstruction may differ according to the causative disease, subgroup analysis in patients with pancreatic cancer was performed. In patients with pancreatic cancer, partially covered SEMSs also had longer TRBO than the other two SEMSs [35–37].

There was no significant difference in complication rate among the three groups. Most of comparative studies have shown no difference in complication rates between covered and uncovered SEMSs [8, 10–13, 19–23]. Nakai et al. reported some risk factors of stent-related cholecystitis [31]. They did not find a significant difference in the incidence of cholecystitis between covered and uncovered SEMSs. The use of covered SEMSs may not be significantly associated with stent-related complications.

There were no significant differences in patient survival among the three groups. Thus, we could avoid any influence of the results from early patient mortality to TRBO. Although obstructions occurred sooner with uncovered and fully covered SEMSs than with partially covered SEMSs, obstructed SEMSs were adequately treated and did not affect patient survival.

This study has several limitations. First, the study population was relatively small and the causative diseases were heterogeneous. Although there were no significant differences in causative diseases among the three groups, the prognosis and tumor progression pattern, which may affect stent patency, vary for each malignancy. Second, this study is retrospective and has potential biases [38, 39].

## Conclusions

Partially covered SEMSs demonstrated longer TRBO than uncovered or fully covered SEMSs. Both partially and fully covered SEMSs prevented tumor ingrowth, which is the most frequent complication of uncovered SEMSs. Importantly, partially covered SEMSs with uncovered flare end succeeded in preventing stent migration, whereas fully covered SEMS with covered flare end did not. Therefore, we recommend the partially covered SEMS with uncovered flare end for patients with MDBO.

## Abbreviations

CI: confidence interval
ERCP: Endoscopic retrograde cholangiopancreatography
HR: hazard ratio
MDBO: malignant distal biliary obstruction
SEMS: Self-expandable metal stent
TRBO: Time to recurrent biliary obstruction
